# The Biogeographic South-North Divide of *Polygonatum* (Asparagaceae Tribe Polygonateae) within Eastern Asia and Its Recent Dispersals in the Northern Hemisphere

**DOI:** 10.1371/journal.pone.0166134

**Published:** 2016-11-03

**Authors:** Jia-Jian Wang, Yong-Ping Yang, Hang Sun, Jun Wen, Tao Deng, Ze-Long Nie, Ying Meng

**Affiliations:** 1 Key Laboratory of Plant Resources Conservation and Utilization, College of Biology and Environmental Sciences, Jishou University, Jishou, Hunan 416000, China; 2 Key Laboratory for Biodiversity and Biogeography of East Asia, Kunming Institute of Botany, Chinese Academy of Sciences, Kunming, Yunnan 650201, China; 3 Department of Botany, National Museum of Natural History, MRC 166, Smithsonian Institution, Washington, DC 20013-7012, United States of America; Austrian Federal Research Centre for Forests BFW, AUSTRIA

## Abstract

Eastern Asia (EA) is a key region for the diversification of flowering plants in the Northern Hemisphere, but few studies have focused on the biogeographic history within EA in the context of the other northern continents. *Polygonatum* is an important medicinal genus widely distributed in the Northern Hemisphere with its highest species richness in EA, and it represents an excellent model for studying the evolution of biogeographic patterns in this region. Divergence time estimation was used to examine the biogeographic history of *Polygonatum* based on nuclear ITS and four plastid sequences (*rbcL*, *matK*, *psbA–trnH* and *trnC–petN*) from 30 *Polygonatum* species and 35 outgroup taxa. The ancestral area of *Polygonatum* and subsequent dispersal routes were inferred using Bayes-Lagrange. *Polygonatum* was estimated to have originated in southern EA during the middle Miocene (14.34–13.57 Ma) with subsequent south-to-north expansion in the late Miocene. Multiple intercontinental dispersal events were inferred between EA and Europe or North America, and all of them have occurred recently in the late Miocene to Pliocene. The separation of *Polygonatum* into the south and north lineages and their subsequent diversifications in the late Miocene supports the existence of a biogeographic divide between the northern and southern parts of EA that also coincides with the retreat and redevelopment of the arid zone in EA in the Neogene. Our results demonstrate the complexity of biogeographic history of *Polygonatum* in the Northern Hemisphere including early vicariance followed by frequent and recent dispersals in the Neogene.

## Introduction

Eastern Asia (EA) is an important region for the biogeographic diversification of flowering plants in the Northern Hemisphere, showing one of the highest levels of species diversity and endemism [1]. It has also been suggested to be the ancestral area for most EA—eastern North American disjunct lineages [2, 3]. The high level of species richness in EA can be attributed to secondary diversification due to habitat heterogeneity as well as a lower rate of extinctions in the Neogene, whereas the lower species diversity in North America and Europe is often explained by the hypothesis of more severe extinctions with global cooling beginning in the late Eocene to Oligocene (Paleogene) [4, 5]. Biogeographic studies on flowering plants in the Northern Hemisphere have largely focused on the classic EA and eastern North American disjunctions, but few studies have been devoted to exploring biogeographic patterns within EA [2, 4, 6]. A better understanding of the biogeographic history of EA and its connections to the other continents, especially concerning the intercontinental disjunctions between EA and North America/Europe, requires the examination of diversification patterns in the Northern Hemisphere from a broader perspective.

*Polygonatum* (commonly known as Solomon’s seal) provides an ideal model for studying the evolution of intercontinental disjunctions in the Northern Hemisphere, as well as the diversifications within EA. The genus contains approximately 60 species that are widely distributed in the warm-temperate to boreal zones of the Northern Hemisphere, and is the largest and most complex genus in the tribe Polygonateae of Asparagaceae [7–9]. Most species of the genus are found in EA (c. 50 species) with two diversification centers: one extending from the Himalayas to southeastern China and the other found in northeastern Asia [10–12]. Apart from these areas, the other species of *Polygonatum* occur in moderate climate zones of North America and Europe, showing a continuous distribution between Europe and Asia and a disjunct distribution between Eurasia and North America. A better understanding of the biogeographic history of *Polygonatum* thus may shed light on the past floristic exchanges between different continents within the Northern Hemisphere, as well as the patterns of biogeographic migrations within EA.

Previous studies have provided insights into the evolutionary relationships of *Polygonatum* [12–16]. Particularly, a recent phylogenetic study based on four plastid markers suggested that three major lineages were well supported in *Polygonatum* [17]. It has been suggested that the lineages showed correlations with geographic distributions, and the phylogenetic results supported the most recent classification at the sectional level. Meng et al. proposed a possible origin of *Polygonatum* in southern EA with subsequent dispersal into northern areas [17].

In this study, we further infer the historical biogeography of *Polygonatum* by employing a Bayesian uncorrelated lognormal and relaxed molecular clock approach based on our previous phylogenetic framework [17]. The ancestral area of *Polygonatum* and subsequent dispersal routes are inferred and compared with the molecular dating results to identify the most likely explanation for the current distribution of the genus. Our study emphasizes elucidating patterns of biogeographic diversification of the genus within EA.

## Materials and Methods

### Ethics Statement

*Polygonatum* species are not included in any Eurasian and North American official list of threatened plants. No special permits were required for this study. The field studies involved no endangered or protected species and the locations of our study are provided in Table 1. Herbarium voucher specimens were deposited in the Kunming Institute of Botany, Chinese Academy of Sciences (KUN) and National Museum of Nature and History (US). The sequences determined in this study are listed in Table 1.

**Table 1:** Information on taxa sampled and corresponding GenBank accession numbers. Accessions beginning with KX are new sequences published in this study; missing data are indicated by a dash. Herbarium acronyms follow http://sciweb.nybg.org.

Taxon	Voucher	Locality	*matK*	*rbcL*	*psbA-trnH*	*trnC-petN*	ITS
*Agave ghiesbreghtii* K. Koch	*––*	*––*	HM640592	HM640478	––	––	––
*Allium microdictyon* Prokh.	*––*	*––*	JF972927	JF972893	––	––	––
*Aphyllanthes monspeliensis* L.	*––*	*––*	JQ276398	JQ273903	––	––	––
*Asparagus* sp.	*Tibet-MacArthur 563* (US)	China: Xizang	––	KX375090	KX375109	––	KX375056
*Behnia reticulata* (Thunb.) Didr.	*––*	*––*	HM640618	HM640500	––	––	––
*Calibanus hookeri* (Lem.) Trel.	*––*	*––*	HM640585	HM640472	––	––	––
*Campylandra fimbriata* (Hand.-Mazz.) M.N. Tamura, S. Yun Liang & Turland	*––*	*––*	HM640562	HM640448	––	––	––
*Convallaria majalis* L.	*Z*.*-L*. *Nie 201* (KUN)	China: Heilongjiang	KJ745641	KJ745528	KJ745890	KJ745987	KX375057
*Dasylirion serratifolium* Baker	*––*	*––*	HM640587	AB029847	––	––	––
*Disporopsis aspersa* (Hua) Engler	*H*. *Li 22773* (KUN)	China: Yunnan	––	KJ745529	KJ745784	KJ745928	KX375058
*Disporopsis fuscopicta* Hance	*Z*.*-L*. *Nie 202* (KUN)	China: Chongqing	KJ745724	KJ745521	KJ745778	KJ745937	KX375059
*Disporopsis longifolia* Craib	*Z*.*-L*. *Nie 2362* (KUN)	China: Guangxi	KJ745662	KJ745582	KJ745836	KJ745944	KX375060
*Disporopsis pernyi* (Hua) Diels	*––*	*––*	HM640566	HM640452	––	––	––
*Dracaena* sp.	*––*	*––*	HM640582	HM640469	GQ435171	––	U24015/U24036
*Eriospermum parvifolium* Jacq.	*––*	*––*	HM640591	HM640477	––	––	––
*Eucomis humilis* Baker	*––*	*––*	HM640624	HM640506	––	––	––
*Heteropolygonatum altelobatum* (Hayata) Y.H. Tseng, H.Y. Tzeng & C.T. Chao	*J*.*-D*. *Chao 1411* (TCF)	China: Taiwan	KJ745742	KJ745592	KJ745789	KJ745975	KX375061
*Heteropolygonatum ginfushanicum* (F.T. Wang & T. Tang) M.N. Tamura, S.C. Chen & Turland	*Z*.*-L*. *Nie 225* (KUN)	China: Chongqing	KJ745698	KJ745621	KJ745782	KJ745938	KX375062
*Heteropolygonatum pendulum* (Z.G. Liu & X.H. Hu) M.N. Tamura & Ogisu	*––*	––	AB029764	AB029831	––	––	––
*Heteropolygonatum roseolum* M.N. Tamura & Ogisu	*Z*.*-L*. *Nie 4077* (KUN)	China: Yunnan	KJ745713	KJ745527	KJ745790	KJ745939	––
*Liriope kansuensis* (Batalin) C.H. Wright	*G*.*-W*. *Hu Z890* (KUN)	China: Jiangxi	KJ745648	KJ745561	KJ745794	KJ745986	KX375063
*Maianthemum bifolium* (L.) F.W. Schmidt	*T*. *Deng 191* (KUN)	China: Heilongjiang	KX375097	KX375091	KX375110	KX375103	KX375064
*Maianthemum henryi* (Baker) LaFrankie	*Z*.*-L*. *Nie 1219* (KUN)	China: Sichuan	KX375098	KX375092	KX375111	KX375104	KX375065
*Maianthemum purpureum* (Wall.) LaFrankie	*Z*.*-L*. *Nie 306* (KUN)	China: Yunnan	KJ745767	KJ745532	KJ745773	KJ745909	KX375066
*Maianthemum racemosum* (L.) Link	*Wen 8562* (US)	USA: Virginia	KJ745667	KJ745533	KJ745772	KJ745906	KX375067
*Maianthemum tatsienense* (Franch.) La Frankie	*Z*.*-L*. *Nie 2099* (KUN)	China: Sichuan	KJ745679	KJ745583	KJ745797	KJ746010	KX375068
*Ophiopogon mairei* H. Léveillé	*Z*.*-L*. *Nie 2372* (KUN)	China: Guangxi	KJ745642	KJ745600	KJ745795	KJ745988	KX375069
*Peliosanthes macrostegia* Hance	*Z*.*-L*. *Nie 3242* (KUN)	China: Yunnan	KJ745647	KJ745599	KJ745796	KJ745904	KX375070
*Polygonatum acuminatifolium* Komarov	*T*. *Deng 108* (KUN)	China: Heilongjiang	KX375099	KX375093	KX375112	KX375105	KX375071
*Polygonatum arisanense* Hayata	*Chen 20110312* (PE)	China: Taiwan	KJ745675	––	––	KJ745967	––
*Polygonatum biflorum* (Walter) Elliott	*T*.*S*. *Yi 20060508_41* (US)	USA: Pennsylvania	KJ745725	KJ745562	KJ745897	KJ745973	KX375072
*Polygonatum cathcartii* Baker	*Tibet-MacArthur 2766* (US)	China: Xizang	KJ745753	KJ745594	KJ745842	––	––
*Polygonatum cirrhifolium* (Wall.) Royle	*Z*.*-L*. *Nie 1148* (KUN)	China: Sichuan	KJ745754	KJ745610	KJ745801	KJ746004	KX375073
*Polygonatum curvistylum* Hua	*Z*.*-L*. *Nie 3045* (KUN)	China: Sichuan	KJ745666	KJ745580	KJ745802	KJ745912	KX375074
*Polygonatum cyrtonema* Hua	*T*. *Deng 146* (KUN)	China: Zhejiang	KJ745644	KJ745523	KJ745785	KJ745949	KX375075
*Polygonatum desoulavyi* Kom.	*––*	Korea	JX903537	JX903128	––	––	––
*Polygonatum falcatum* A. Gray	*––*	Korea	JX903538	JX903129	––	––	––
*Polygonatum filipes* Merrill ex C. Jeffrey & McEwan	T. Deng *53* (KUN)	China: Zhejiang	KX375100	KX375094	KX375113	KX375106	KX375076
*Polygonatum franchetii* Hua	*T*. *Deng HJ13* (KUN)	China: Hunan	––	KJ745558	KJ745833	KJ745977	––
*Polygonatum grandicaule* Y.S. Kim, B.U. Oh & C.G. Jang	PDBK2010-1721	Korea	KC704686	KC704944	KC704416	––	––
*Polygonatum griffithii* Baker	*Tibet-MacArthur 615* (US)	China: Xizang	KJ745752	KJ745552	KJ745781	KJ745926	KX375077
*Polygonatum hirtellum* Handel-Mazzetti	*Z*.*-L*. *Nie 2839* (KUN)	China: Sichuan	KJ745758	KJ745541	KJ745822	KJ745916	––
*Polygonatum hookeri* Baker	*Z*.*-L*. *Nie 2994* (KUN)	China: Sichuan	KJ745699	KJ745622	KJ745811	KJ745923	KX375078
*Polygonatum humile* Fischer ex Maximowicz	*T*. *Deng 107* (KUN)	China: Heilongjiang	KJ745702	KJ745612	KJ745862	KJ745955	KX375079
*Polygonatum inflatum* Komarov	*T*. *Deng 113* (KUN)	China: Heilongjiang	KJ745685	KJ745524	KJ745856	KJ745954	KX375080
*Polygonatum involucratum* (Franchet & Savatier) Maximowicz	*T*. *Deng 125* (KUN)	China: Heilongjiang	KX375101	KX375095	KX375114	KX375107	KX375081
*Polygonatum kingianum* Collett & Hemsley	*Z*.*-L*. *Nie 729* (KUN)	China: Yunnan	KJ745691	KJ745517	KJ745831	KJ745941	KX375082
*Polygonatum lasianthum* Maxim.	*––*	Korea	HM640572	HM640458	––	––	––
*Polygonatum multiflorum* (L.) All.	*Junheil sn*	Germany: Lahngergo	KJ745690	––	KJ745776	KJ746009	KX375083
*Polygonatum odoratum* (Mill.) Druce	*Wen 10321* (US)	Russia	KJ745674	KJ745630	KJ745852	KJ745911	––
*Polygonatum oppositifolium* (Wall.) Royle	*––*	*––*	AB029763	AB029830	––	––	––
*Polygonatum prattii* Baker	*Z*.*-L*. *Nie 2130* (KUN)	China: Guizhou	KJ745712	KJ745624	KJ745815	KJ745915	––
*Polygonatum pubescens* (Willd.) Pursh	*Z*.*-L*. *Nie 523* (KUN)	USA: Virginia	KJ745722	KJ745534	KJ745896	KJ745972	KX375084
*Polygonatum punctatum* Royle ex Kunth	*G*.*-W*. *Hu 402* (KUN)	China: Yunnan	KJ745678	KJ745554	KJ745798	KJ746008	––
*Polygonatum robustum* Nakai	PDBK2012-0257	Korea	KC704714	KC704960	KC704444	––	––
*Polygonatum roseum* (Ledebour) Kunth	*Wen 10365* (US)	Russia	KJ745676	KJ745631	KJ745806	KJ745922	––
*Polygonatum sibiricum* Redouté	*T*. *Deng 165* (KUN)	China: Anhui	KJ745706	KJ745581	KJ745880	KJ745931	KX375085
*Polygonatum* sp.	*Wen10371* (US)	Russia:	KX375102	KX375096	KX375115	KX375108	––
*Polygonatum stenophyllum* Maxim.	*Z*.*-L*. *Nie 695* (KUN)	China: Helongjiang	KJ745763	KJ745556	KJ745894	KJ745964	KX375086
*Polygonatum verticillatum* (L.) All.	*Tibet-MacArthur 3003* (US)	China: Xizang	KJ745735	KJ745596	KJ745840	––	KX375087
*Polygonatum zanlanscianense* Pamp.	*T*. *Deng 054* (KUN)	China: Zhejiang	KJ745755	KJ745608	KJ745820	KJ745921	KX375088
*Reineckea carnea* (Andrews) Kunth	*Z*.*-L*. *Nie 2172* (KUN)	China: Guizhou	KJ745649	KJ745579	KJ745791	KJ745907	KX375089
*Ruscus aculeatus* L.	*––*	*––*	HM640554	HM640440	––	––	––
*Sansevieria trifasciata* Prain	*––*	*––*	HM640584	HM640471	FN675812	––	U23992/U24050
*Scilla scilloides* Druce	*––*	*––*	HM640632	HM640514	––	––	––
*Speirantha gardenii* (Hook.) Baill.	*T*. *Deng 012* (KUN)	China: Anhui	KJ745743	KJ745584	KJ745891	KJ745908	––
*Theropogon pallidus* Maxim.	*––*	*––*	HM640560	HM640446	––	––	––
*Tricalistra ochracea* Ridl.	*––*	*––*	AB029777	AB029839	––	––	––

### Taxon sampling and sequence dataset

A total of 68 accessions representing 33 species of *Polygonatum* were included in the analyses (Table 1). Our sampling of the ingroup covers the geographic diversity of the genus (28 of over 50 recognized species in EA, three of the five species from Europe, and the two species from North America), and represents a wide range of morphological forms from all sections [12] and the eight series [9]. The outgroup taxa were selected to include representatives from other genera in tribe Polygonateae (i.e., *Heteropolygonatum*, *Disporopsis*, and *Maianthemum*; 13 species) and the other members in Asparagaceae (21 species) based on previous broader analyses [18–20]. *Allium microdictyon* from Amaryllidaceae was included in the outgroup to enable rooting of the tree topology and calibration of the molecular clock.

An existing dataset from two plastid coding regions (*rbcL* and *matK*) and two non-coding regions (*psbA-trnH* and *trnC-petN*) have been supplemented with new sequences for a few additional species (Table 1), using methodologies described in our earlier publications [17, 21]. Sequences for four plastid DNA regions were obtained from GenBank (with most of the ingroup sequences derived from our previous studies [17,21]). To provide better resolution with evidence from the nuclear genome, an internal transcribed spacer (ITS) dataset of 32 species (18 *Polygonatum* and 14 outgroup) was newly generated in our study. The entire ITS region was amplified and sequenced using the ITS1 (occasionally using ITS5) and ITS4 primers [22]. When amplification of the ITS region was unsuccessful, two internal primers ITS2 and ITS3 [23] were used to obtain PCR products in two shorter fragment (ITS1—ITS2, ITS3—ITS4). The ITS sequences were amplified and sequenced following Meng et al. [21]. Information on the voucher specimens used and the corresponding GenBank accession numbers are provided in Table 1. Sequence alignment was performed in MAFFT 6 using the default alignment parameters [24] followed by manual adjustment in PhyDE 1.0 [25].

### Bayesian time estimations

Independent DNA substitution model and gamma rate heterogeneity were estimated on each molecular marker using the Akaike Information Criterion as determined by MrModelTest 2.3 (Akaike 1974; Nylander 2008). The HKY+G+I model was selected for *rbcL* and *psbA-trnH*; the GTR+G model was selected for *matK* and ITS; and the GTR+G+I model was selected for *petN-trnC*. For the molecular dating analyses, the strict molecular clock model was rejected (P <0.01) for our dataset based on a likelihood ratio test [26]. We simultaneously estimated the tree topology and node ages of *Polygonatum* using a Bayesian relaxed clock model as implemented in BEAST 1.8.0 [27].

The molecular sequence fragments were partitioned using BEAUti 1.8.0 (as supplied within BEAST program) in order to incorporate different models of substitution rates for each partition separately. A Yule speciation tree prior was specified using an uncorrelated lognormal distributed (UCLD) relaxed clock model [28]. After optimal operator adjustment as suggested by the output diagnostics from the preliminary BEAST runs, two independent Markov Chain Monte Carlos (MCMC) runs were performed for 50 million generations each and sampling every 1000 generations. Tracer 1.6 [29] was used to check for convergence between the runs. The results are considered reliable once the effective sampling size for all parameters exceeded 200, as suggested by the program manual [29]. After the removal of the 10% burn-in, the sampled posterior trees were summarized to generate a maximum clade credibility tree using the program TreeAnnotator 1.8.0 [27] with a posterior probabilities (PP) limit of 0.5 and mean node heights.

Calibrations of molecular phylogenetic trees are generally better when performed using multiple fossil records [30]. Due to the fact that there are no reliable fossils assigned to Asparagaceae [31], two secondary-calibration points were used in our analyses, following the approach described in recent studies on Tecophilaeaceae and Asparagaceae subfamily Scilloideae [32, 33]. First, the most recent common ancestor of Asparagaceae and the outgroup taxa were constrained using a normal distribution with a mean of 58.3 million years ago (Ma) and a standard deviation of six. Secondly, the crown node of the family was constrained with a normal distribution, a mean of 56.4 Ma with a standard deviation of six. These values were obtained from previous molecular estimates [18, 34] and were used to calibrate the BEAST analysis. Based on fossil calibrations from all monocots, Chen et al. [18] reported the following divergence times for Asparagaceae: a crown age of 56.4 (48.1–65.3) Ma and a stem age of 58.3 (49.9–67.4) Ma. Bell et al. [34] suggested a crown age of 51 (42–59) to 56 (47–66) Ma and a stem age of 54 (45–62) to 60 (52–69) Ma for Asparagaceae.

To minimize the effects from the secondary-calibrations, an additional time estimation was also implemented based on a large dataset of *rbcL* and *matK* sequences which sampled 245 taxa representing all monocots to enable multiple fossil constraints (S1 Table). We constrained the crown Pandanales, with a minimum age (89.3 Ma) corresponding to the oldest known fossil records from *Mabelia* and *Nuhliantha* [35]. We also used fossil evidences to set the minimum stem age of Arecales at 70.6 Ma and the stem of Zingiberales at 72.5 Ma, as discussed in Hertweck et al. [36]. A minimum age of 14.5 Ma was fixed at the stem age of *Yucca* based on the fossil records from *Protoyucca shadishii* [37, 38]. With the chloroplast phylogenetic framework and multiple fossil calibrations, the split between *Acorus* and the remaining monocots was estimated to be 131 (126–142) or 138 (132–143) Ma using the penalized likelihood or relaxed clock approaches, respectively [37]. We thus constrained the root of our tree to 134.5 +/- 5.0 and the maximum ages of all the fixed points as 134.5 Ma using uniform priors in our analyses.

### Ancestral area reconstruction

The distribution range of *Polygonatum* and its sister taxa was divided into three areas, based on the geographic boundary and the presence of one or more endemic species. These areas are: A (eastern Asia, EA), B (North America), and C (Europe to central Asia). We also ran a second analysis with EA split into the southern (A) and the northern regions (D) in the biogeographic analysis, because *Polygonatum* has its highest species diversity and endemism in these two regions [7, 9–11]. Eastern Asia can be subdivided into two distinct northern and southern regions [3, 6, 39–41]. Geological evidences suggest that an aridity barrier has existed from the western-most part of China to the eastern Asian coast from the Paleogene to the Miocene, and it has been thought to have acted as a climate barrier between the two regions [42]. The arid belt around 35° N in China [43] was used as a boundary to define these two regions in this study. The southern region of EA (sEA) comprises southern and southeastern China, with extensions into the Himalayas, whereas the northern region of EA (nEA) comprises northeast China, Korea and Japan.

A likelihood method for biogeographic inference was applied in Lagrange 20130526 [44], which take into account genetic branch lengths and/or phylogenetic uncertainty and incorporates an explicit dispersal-extinction-cladogenesis (DEC) model of dispersal routes available at historical intervals correlating stochastic events with lineage persistence [44]. We used a recently modified statistic DEC (S-DEC) analysis implemented in RASP 3.2 to reconstruct the possible ancestral ranges of *Polygonatum* on the phylogenetic trees [45]. To account for uncertainties in phylogeny and DEC optimizations, we used 1000 randomly sampled trees from the post burn-in sampled trees derived from the first BEAST analysis. The 40 taxa in the *Polygonatum–Disporopsis*–*Heteropolygonatum* clade were included with the other taxa and pruned from the input trees. The Bayes-Lagrange analysis [45, 46] was then performed in RASP on all of the trees to obtain the possible ancestral range at each node.

## Results

**Fig 1:**
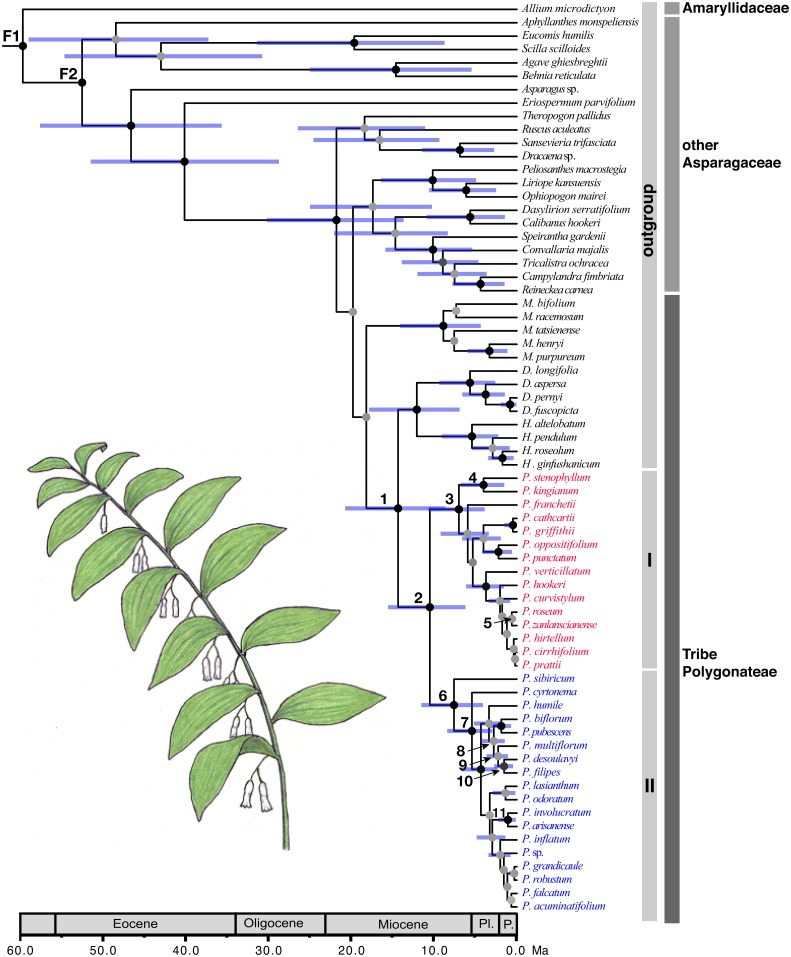
Maximum clade credibility tree of *Polygonatum* and the closely related taxa derived from a BEAST analysis. Posterior estimates of divergence times were inferred using a partitioned analysis of five combined DNA regions (ITS, *psbA-trnH*, *trnC-petN*, *rbcL*, and *matK*) and two fixes as normal age constraints (F1: 58.3 Ma; F2: 56.4 Ma). Nodes are posterior mean ages with blue node bars representing 95% highest posterior density intervals. Bayesian posterior probabilities <0.95 are indicated by grey and >0.95 by black circles. Geological epoch abbreviations: Pl, Pliocene; P, Pleistocene. Inset: *Polygonatum pubescens*.

The final datasets of *rbcL*, *matK*, *psbA–trnH*, *trnC–petN*, and ITS consist of 1447 (224 variable and 110 potentially parsimony-informative), 1864 (558 variable and 245 potentially parsimony-informative), 729 (56 variable and 21 potentially parsimony-informative), 985 (96 variable and 42 potentially parsimony-informative), and 741 (256 variable and 136 potentially parsimony-informative) nucleotide positions, respectively. The total number of characters included in the combined dataset is 5766 nucleotides. The BEAST analysis produced a well-resolved phylogeny of *Polygonatum*, which strongly supported the monophyly of *Polygonatum* and two major clades within the genus (Fig 1). *Polygonatum biflorum* and *P*. *pubescens* from eastern North America are deeply nested within sect. *Polygonatum*, showing a close relationship to the Eurasian clade including *P*. *multiflorum*, *P*. *filipes* and *P*. *desoulavyi*.

The Bayesian dating analyses based on two different datasets and different calibration schemes produced similar results (Table 2, see also S1 Fig). An origin of the *Polygonatum* stem lineage was estimated to be 14.34 Ma with a 95% highest posterior density (HPD) range of 8.64–20.74 Ma (node 1, Fig 1) or 13.57 (9.13–18.42) Ma based on the two calibration schemes (Table 2, S1 Fig). The initial diversification of the *Polygonatum* crown group was inferred to be 10.5 (6.21–15.54; node 2 in Fig 1) or 9.85 (6.3–13.65) Ma. The disjunction between Eurasian and North American species was estimated at 2.79 (1.46–4.38; Fig 1) or 3.31 (1.65–5.18) Ma. Details of ages for other nodes of interest are shown in Table 1.

**Table 2:** Posterior age distributions of major nodes of *Polygonatum* (Asparagaceae), with results of ancestral area reconstruction using the S-DEC. Node numbers correlate with those in Fig 2.

	Age estimate 1		Age estimate 2		Ancestral area reconstruction	
Node	Mean (Ma)	95% HPD (Ma)	Mean (Ma)	95% HPD (Ma)	S-DEC 1	S-DEC 2
1	14.34	8.64–20.74	13.57	9.13–18.42	A 100%	A 55%, AD 41%
2	10.5	6.21–15.54	9.85	6.3–13.65	A 100%	A 52%, AD 36%
3	7.0	3.89–10.66	7.08	3.99–10.41	A 100%	A 68%, AD 32%
4	4.02	1.52–10.66	––	––	A 100%	AD 100%
5	0.61	0.09–1.27	0.45	0–1.19	AC 100%	AC 100%
6	7.59	4.1–11.53	7.86	4.98–11.32	A 100%	AD 62%, D 21%
7	5.42	2.98–8.4	––	––	A 100%	AD 67%
8	2.79	1.46–4.38	3.31	1.65–5.18	AB 78%, A 20%	BCD 40%, ABD 31%
9	2.25	1.08–3.67	––	––	AC 69%, A 30%	ACD 61%, CD 28%
10	1.54	0.46–2.74	1.84	0.53–3.31	A 100%	AD 100%
11	1.08	0.13–2.24	––	––	A 100%	AD 100%

**Fig 2:**
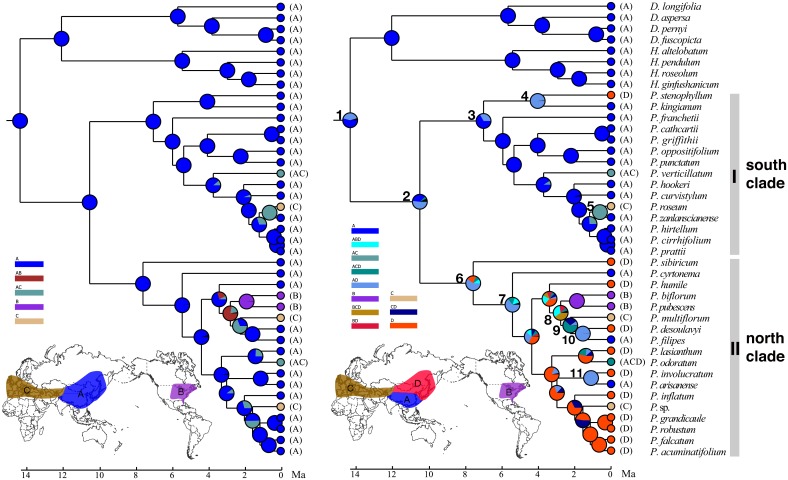
Reconstruction of ancestral distributions in *Polygonatum* using Bayes-Lagrange optimizations. Pie charts at nodes represent relative frequencies of ancestral-area reconstructions. The letter and color-coding of regions for each tree is indicated on the map. Numbers 1–11 indicate nodes of interest (see Table 2 for details).

The ancestral distributions inferred from S-DEC for internal nodes in the genus are shown in Fig 2 and Table 2. The first analysis revealed that *Polygonatum* originated in EA with several migrations or dispersals into other regions of the Northern Hemisphere. The second S-DEC analysis indicated two possible ancestral areas for node 1, A (sEA) or AD (both sEA and nEA), and the frequencies of occurrence of these ranges are 55% and 41%, respectively (Fig 2; Table 2). A similar result was found for the crown lineage of the genus (node 2, Fig 2), the north and the south clades (Fig 2; Table 2). A recent migration from sEA to central Asia was found in *P*. *roseum* with its sister taxon (*P*. *zanlanscianense*), with a divergence time of 0.61 to 0.45 Ma (node 5, Fig 2; Table 2). Dispersal into central Asia and Europe was also observed in *P*. *multiflorum* and its sister taxa (*P*. *filipes* and *P*. *desoulavyi*), which diverged at 2.25 Ma (node 9; Fig 2; Table 2).

## Discussion

### Early divergence within EA

The BEAST analysis produced a robust phylogeny of *Polygonum* that is congruent with our previous phylogenetic results [17] and suggested an origin of the genus in the middle Miocene around 14.34 or 13.57 Ma (Fig 2). *Polygonatum* together with its sister group (*Disporopsis* and *Heteropolygonatum*) are mainly restricted or endemic to EA [9, 10, 12] and our first S-DEC analysis also suggested EA as the ancestral area of the genus (Fig 2; Table 2). The results from the second S-DEC further revealed that sEA is more likely to be the ancestral range of the stem lineage of *Polygonatum* (A, 55%; node 1 in Fig 2), although EA (sEA + nEA) is also possible (AD, 41%). The favored ancestral range at the crown lineage of *Polygonatum* (node 2, Fig 2) is also sEA (A, 52%; Table 2), which suggested an early origin and differentiation of the genus in this area.

This early south to north colonization within EA resulted in a split of *Polygonatum* into two major lineages in the late Miocene (10.5 to 9.85 Ma; node 2 in Fig 1): one is the southern clade (clade I, Fig 2) including approximately 50% of the species in the genus distributed from southeastern China to the eastern Himalayan region; and the other (clade II, Fig 2) comprising most of the remaining species, which are primarily distributed from the northeastern part of the Sino-Japanese region.

The separation of *Polygonatum* into the southern and the northern lineages and their subsequent diversifications in the late Miocene (node 2, Fig 2) is probably within a time frame coinciding with paleoclimatic events in EA. As suggested by palynological, paleobotanical, and lithologic data, the Paleogene in continental Asia might inherit the environmental pattern of the late Cretaceous with a broad arid zone from its west to east [42, 47], which may have served as a possible migration barrier for *Polygonatum* from southern to northern regions within EA. In the Miocene, the arid zone retreated to northwestern China, and eastern China became more humid because of the strengthening of the southeast summer monsoon [47–50], which perhaps provided open routes for *Polygonatum* to colonize new habitats in more northern areas. Yet again, as the late Miocene progressed (c. 11–5.3 Ma), the arid belt might have redeveloped and almost reached the coast of northern China towards the Pliocene [42]. As a consequence, this arid belt could have acted as a climate barrier that impeded gene flow between the Neogene relict plants in two EA regions and promoted their diversification in the late Miocene to Pliocene, as indicated by the two lineages in *Polygonatum* (Fig 2). However, to our knowledge, there have been very few biogeographic or phylogeographic studies to test the existence of a biogeographic divide between northern and southern EA during the Miocene and into the Pliocene, except for among Asian butternuts (*Juglans* section *Cardiocaryon*), which offer another case supporting this hypothesis [43]. The genetic data from both chloroplast and nuclear regions consistently identified two clades (one northern and one southern) in the Asian butternuts, which diverged through climate-induced vicariance during the mid-Miocene [43].

Based on a sEA origin of the genus, a south to north expansion is suggested for the biogeographic origin of the northern clade within EA (AD, 62%, node 6 in Fig 2). However, plant migrations within EA have been proposed to be primarily in a north to south direction [51–53], which has been supported by molecular evidence from many angiosperm taxa. For example, *Maianthemum* is the second largest genus after *Polygonatum* in tribe Polygonateae and exhibits a similar distribution pattern to that of *Polygonatum* in the Northern Hemisphere. However, most *Maianthemum* species from the Himalayas to southwestern China form a well supported clade nested within several basal grades, including species from nEA and the New World [21], which may indicate a possible New World origin and a north to south migration within EA. *Astilbe* Buch.-Ham. ex D.Don is a well-known genus of Saxifragaceae with an early divergence in nEA, which then migrated southwards into southern and tropical Asia [54]. It is also well supported that *Phryma* L. (Phrymaceae) diversified in nEA first and then migrated to sEA [55]. In contrast, the biogeographic diversification of *Polygonatum* in EA represents one of the few examples showing a south to north migration scenario. Notably, some plant lineages have been reported to have southern or tropical origins with adaptations to northern and seasonal climates in EA [56], especially for north temperate taxa with tropical affinities such as *Mitchella* L. (Rubiaceae) [57] and *Parthenocissus* Planch. (Vitaceae) [58].

### Recent dispersals in shaping modern distribution

Our biogeographic results revealed multiple recent migrations/exchanges among areas of EA, Europe (including central Asia), and North America from the late Miocene to the Pliocene (Fig 2). The divergence time between Eurasia (*Polygonatum multiflorum*, *P*. *filipes* and *P*. *desoulavyi*) and eastern North America (*P*. *biflorum* and *P*. *pubescens*) was estimated to be 2.79 to 3.31 Ma in the Pliocene (node 8, Fig 1; Table 2). The biogeographic inference supported a migration from EA into North America (Fig 2). The biogeographic disjunctions in EA and North America are common in plants and have been explained by either vicariance (via the Bering or the North Atlantic land bridges) or long-distance dispersal [2–4, 59]. The Bering land bridge was available for floristic exchanges until 3.5 to 5.0 Ma in the late Tertiary [60], and thus appears to be the most likely migration route for *Polygonatum*. Beringian migrations have been proposed for a number of flowering plant taxa, especially for herbs with recent disjunct ages, including *Circaea* L. (Onagraceae) [61], *Symplocarpus* R.A.Salisbury ex Nuttall (Araceae) [62], and *Phryma* (Phrymaceae) [55]. The biogeographic disjunction between the North American and European and Asian alternate-leaved taxa suggest that there is a possibility that the North Atlantic land bridge might have been a viable pathway, given the absence of *Polygonatum* in most of western North America. However, the North Atlantic route has been discussed as a more likely route for thermophilic and woody taxa available before the Oligocene [63] and seems less likely in our case.

The other biogeographic connections are found between Europe to central Asia and EA, as indicated by many taxa from central Asia and Europe nested with taxa from EA (Fig 2). For example, Pliocene migrations or dispersals from EA into central Asia and Europe are found between *P*. *roseum* and its sister taxon (*P*. *zanlanscianense*) and between *P*. *multiflorum* and its sister taxon (*P*. *filipes* and *P*. *desoulavyi*) (Fig 2; Table 2). Since the late Eocene, the Turgai Strait retreated southward, coinciding with the global cooling of the Eocene-Oligocene boundary. This retreat allowed extensive biogeographic exchange between Asia and Europe [50]. The wide spread of *P*. *verticillatum*, from Europe to EA, may be another example showing this biogeographic connection (Fig 2).

Recent exchanges between southern and northern regions within EA were also observed in the late Miocene and Pliocene. The first example is the northern species of *P*. *stenophyllum* that diverged from its relative *P*. *kingianum* from subtropical SW China to Indochina at 4.02 Ma (node 4, Fig 2; Table 2). The southern species of *P*. *filipes* from eastern China (node 10, Fig 2; Table 2) and *P*. *arisanease* from Taiwan (node 11, Fig 2; Table 2) show the opposite migration route (from north to south) at 1.54–1.84 and 1.08 Ma with their sister species *P*. *desoulavyi* and *P*. *involucratum* from northeastern Asia, respectively.

In a word, all the disjunct dispersals or migrations in *Polygonatum*, either out of EA to Europe or North America, or within EA (e.g., from the southern to the northern part, or vice versa), are much recent and mostly restricted to the late Miocene to Pliocene (Fig 2). It seems that these recent dispersals finally led to a widespread distribution of the *Polygonatum* in the Northern Hemisphere. Similar pattern of recent dispersals have been found in various flowering plants which indicated the importance of the late Miocene–Pliocene in the assembly of the modern flora [64].

## Conclusions

*Polygonatum* likely originated in sEA with a subsequent split into southern and northern lineages, supporting the existence of a biogeographic divide between the northern and the southern parts of EA during the Miocene. This biogeographic subdivision is probably explained by the retreat and redevelopment of the arid zone in EA in the Neogene. Plant migrations within EA have been proposed to have primarily occurred in a north to south direction; however, the biogeographic history of *Polygonatum* in EA documents primarily a south to north migration scenario. Our results also indicate that the modern distribution of *Polygonatum* in the Northern Hemisphere has resulted from recent regional dispersals from EA to North America, central Asia, and Europe during the late Miocene to Pliocene.

## Supporting Information

S1 Fig Maximum clade credibility tree of *Polygonatum* based on a broad sampling of 245 monocot taxa. Posterior estimates of divergence times were inferred using a partitioned analysis of two combined plastid DNA regions (*rbcL* and *matK*) and fossil-based calibrations (1–5). Nodes are posterior mean ages with blue node bars representing 95% highest posterior density intervals.
(PDF)

S1 Table Taxa sampled from the whole monocots with GenBank numbers used in our second time estimation. (DOCX)
