# Supplementary figures and images for: The Biogeographic South-North Divide of Polygonatum (Asparagaceae Tribe Polygonateae) within Eastern Asia and Its Recent Dispersals in the Northern Hemisphere
Source: PLoS One. 2016 Nov 3;11(11):e0166134. doi: 10.1371/journal.pone.0166134 (PMC5094755; doi:10.1371/journal.pone.0166134)

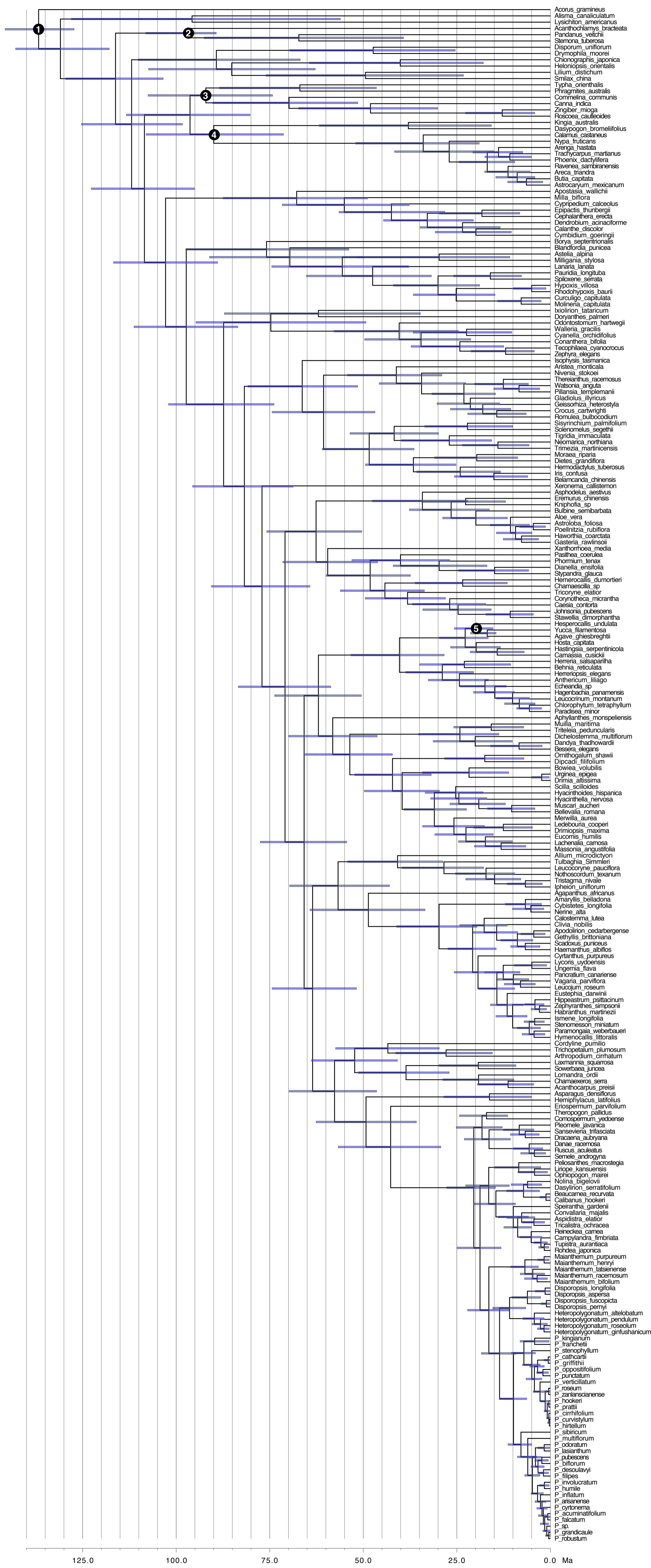

Supplement: S1 Fig — Posterior estimates of divergence times were inferred using a partitioned analysis of two combined plastid DNA regions (rbcL and matK) and fossil-based calibrations (1–5). Nodes are posterior mean ages with blue node bars representing 95% highest posterior density intervals. (PDF) [file pone.0166134.s001.pdf]
