# Supplementary material for: The Biogeographic South-North Divide of Polygonatum (Asparagaceae Tribe Polygonateae) within Eastern Asia and Its Recent Dispersals in the Northern Hemisphere
Source: PLoS One. 2016 Nov 3;11(11):e0166134. doi: 10.1371/journal.pone.0166134 (PMC5094755; doi:10.1371/journal.pone.0166134)
Supplement: S1 Table — (DOCX) [file pone.0166134.s002.docx]

| Table S1 Taxa sampled from the whole monocots with GenBank numbers used in our second time estimation. | | |
| --- | --- | --- |
| Taxa | *rbcL* | *matK* |
| *Acanthocarpus preisii* | JX903182 | JX903591 |
| *Acorus gramineus* | KP099646 | KP099646 |
| *Agapanthus africanus* | HM640485 | HM640599 |
| *Agave ghiesbreghtii* | HM640478 | HM640592 |
| *Allium microdictyon* | JF972893 | JF972927 |
| *Aloe vera* | AJ512309 | AJ511390 |
| *Amaryllis belladona* | JX903144 | JX903555 |
| *Anthericum liliago* | HM640490 | HM640605 |
| *Aphyllanthes monspeliensis* | JF972914 | HM640614 |
| *Apodolirion cedarbergense* | JX903145 | JX903556 |
| *Apostasia wallichii* | HM640552 | AY557212 |
| *Areca triandra* | JX903249 | AM114664 |
| *Arenga hastata* | JX903250 | JX903666 |
| *Aristea monticala* | JX903212 | JX903622 |
| *Arthropodium cirrhatum* | HM640516 | HM640634 |
| *Asparagus densiflorus* | JX903171 | JX903580 |
| *Asphodelus aestivus* | HM640527 | HM640645 |
| *Aspidistra elatior* | HM640447 | HM640561 |
| *Astelia alpina* | HM640530 | HM640648 |
| *Astrocaryum mexicanum* | JX903251 | JX903667 |
| *Astroloba foliosa* | JX903193 | JX903601 |
| *Beaucarnea recurvata* | JX903131 | JX903542 |
| *Behnia reticulata* | HM640500 | HM640618 |
| *Belamcanda chinensis* | JF972897 | JF972931 |
| *Bellevalia romana* | JX903174 | JX903583 |
| *Bessera elegans* | HM640519 | HM640637 |
| *Blandfordia punicea* | HM640532 | HM640650 |
| *Borya septentrionalis* | HM640533 | HM640651 |
| *Bowiea volubilis* | HM640503 | HM640621 |
| *Bulbine semibarbata* | HM640528 | HM640646 |
| *Butia capitata* | JX903252 | JX903668 |
| *Caesia contorta* | JX903201 | JX903610 |
| *Calamus castaneus* | M81810 | JX903669 |
| *Calanthe discolor* | HM640548 | HM640665 |
| *Calibanus hookeri* | HM640472 | HM640585 |
| *Calostemma lutea* | JX903146 | JX903557 |
| *Camassia cusickii* | HM640479 | HM640593 |
| *Campylandra fimbriata* | HM640448 | HM640562 |
| *Canna indica* | JX903257 | JX903674 |
| *Cephalanthera erecta* | JF972908 | JF972942 |
| *Chamaescilla sp* | JX903183 | JX903592 |
| *Chamaexeros serra* | JX903184 | JX903593 |
| *Chionographis japonica* | JX903242 | JX903658 |
| *Chlorophytum tetraphyllum* | JX903169 | HM640609 |
| *Clivia nobilis* | JX903147 | HM640603 |
| *Commelina communis* | JX903248 | JX903665 |
| *Comospermum yedoense* | HM640494 | HM640610 |
| *Conanthera bifolia* | JX903230 | JX903646 |
| *Convallaria majalis* | HM640443 | HM640557 |
| *Cordyline pumilio* | JX903186 | JX903595 |
| *Corynotheca micrantha* | JX903202 | JX903611 |
| *Crocus cartwrighti* | JX903214 | JX903624 |
| *Curculigo capitulata* | HM640538 | HM640656 |
| *Cyanella orchidifolius* | HM640545 | HM640662 |
| *Cybistetes longifolia* | JX903148 | JX903558 |
| *Cymbidium goeringii* | JF972910 | JF972944 |
| *Cypripedium calceolus* | HM640550 | HM640667 |
| *Cyrtanthus purpureus* | JX903149 | JX903559 |
| *Danae racemosa* | HM640439 | HM640553 |
| *Dandya thadhowardii* | HM640521 | HM640639 |
| *Dasylirion serratifolium* | AB029847 | HM640587 |
| *Dendrobium acinaciforme* | *FJ216578 | JX903644 |
| *Dianella ensifolia* | HM640536 | HM640654 |
| *Dichelostemma multiflorum* | HM640522 | HM640640 |
| *Dietes grandiflora* | JX903215 | JX903625 |
| *Dipcadi filifolium* | HM640504 | HM640622 |
| *Disporopsis aspersa* | KJ745529 | -- |
| *Disporopsis fuscopicta* | KJ745521 | KJ745724 |
| *Disporopsis longifolia* | KJ745582 | KJ745662 |
| *Disporopsis pernyi* | HM640452 | HM640566 |
| *Disporum uniflorum* | JX903237 | JX903653 |
| *Doryanthes palmeri* | HM640535 | HM640653 |
| *Dracaena aubryana* | HM640470 | HM640583 |
| *Drimia altissima* | HM640505 | HM640623 |
| *Drimiopsis maxima* | JX903175 | JX903584 |
| *Drymophila moorei* | JX903241 | JX903657 |
| *Echeandia sp* | HM640495 | HM640612 |
| *Epipactis thunbergii* | JF972911 | JX903645 |
| *Eremurus chinensis* | HM640526 | HM640644 |
| *Eriospermum parvifolium* | HM640477 | HM640591 |
| *Eucomis humilis* | HM640506 | HM640624 |
| *Eustephia darwinii* | JX903150 | JX903560 |
| *Gasteria rawlinsoii* | JX903195 | JX903603 |
| *Geissorhiza heterostyla* | JX903216 | JX903626 |
| *Gethyllis brittoniana* | JX903151 | JX903561 |
| *Gladiolus illyricus* | HM640542 | JX903627 |
| *Habranthus martinezii* | JX903152 | JX903562 |
| *Haemanthus albiflos* | JX903153 | JX903563 |
| *Hagenbachia panamensis* | JX903170 | JX903579 |
| *Hastingsia serpentinicola* | JX903177 | JX903586 |
| *Haworthia coarctata* | JX903196 | JX903604 |
| *Heloniopsis orientalis* | JX903243 | JX903659 |
| *Hemerocallis dumortieri* | JF972904 | JF972938 |
| *Hemiphylacus latifolius* | HM640499 | HM640617 |
| *Hermodactylus tuberosus* | JX903217 | JX903628 |
| *Herreria salsaparilha* | HM640501 | HM640619 |
| *Herreriopsis elegans* | JX903172 | JX903581 |
| *Hesperocallis undulata* | HM640502 | HM640620 |
| *Heteropolygonatum altelobatum* | KJ745592 | KJ745742 |
| *Heteropolygonatum ginfushanicum* | KJ745621 | KJ745698 |
| *Heteropolygonatum pendulum* | AB029831 | AB029764 |
| *Heteropolygonatum roseolum* | KJ745527 | KJ745713 |
| *Hippeastrum psittacinum* | JX903155 | JX903565 |
| *Hosta capitata* | JF972890 | JF972924 |
| *Hyacinthella nervosa* | JX903178 | JX903587 |
| *Hyacinthoides hispanica* | JX903179 | JX903588 |
| *Hymenocallis littoralis* | JX903156 | JX903566 |
| *Hypoxis villosa* | JX903208 | JX903619 |
| *Ipheion uniflorum* | HM640484 | HM640598 |
| *Iris confusa* | JX903218 | JX903629 |
| *Ismene longifolia* | JX903157 | JX903567 |
| *Isophysis tasmanica* | JX903219 | JX903630 |
| *Ixiolirion tataricum* | HM640543 | HM640660 |
| *Johnsonia pubescens* | JX903203 | JX903613 |
| *Kniphofia sp* | Z73689 | JX903605 |
| *Lachenalia carnosa* | HM640507 | HM640625 |
| *Lanaria lanata* | JF972916 | JF972948 |
| *Laxmannia squarrosa* | HM640518 | HM640636 |
| *Ledebouria cooperi* | HM640508 | HM640626 |
| *Leucocoryne pauciflora* | JX903137 | JX903548 |
| *Leucocrinum montanum* | HM640481 | HM640595 |
| *Leucojum roseum* | JX903158 | JX903568 |
| *Lilium distichum* | JX903238 | JX903654 |
| *Liriope kansuensis* | KJ745648 | KJ745561 |
| *Lomandra ordii* | JX903188 | JX903596 |
| *Lycoris uydoensis* | HM640486 | HM640600 |
| *Maianthemum bicolor* | HM640460 | HM640574 |
| *Maianthemum bifolium* | KJ745531 | KJ745660 |
| *Maianthemum dahurica* | HM640461 | HM640575 |
| *Maianthemum dilatatum* | HM640454 | HM640568 |
| *Maianthemum henryi* | KJ745620 | KJ745714 |
| *Maianthemum japonica* | HM640462 | HM640576 |
| *Maianthemum purpureum* | KJ745532 | KJ745767 |
| *Maianthemum racemosum* | KJ745533 | KJ745667 |
| *Maianthemum stellatum* | JX903127 | JX903536 |
| *Maianthemum tatsienense* | KJ745583 | KJ745679 |
| *Massonia angustifolia* | HM640509 | HM640627 |
| *Merwilla aurea* | JX903180 | JX903589 |
| *Milla biflora* | HM640523 | HM640641 |
| *Milligania stylosa* | HM640531 | HM640649 |
| *Molineria capitulata* | JX903209 | AB088783 |
| *Moraea riparia* | JX903220 | JX903631 |
| *Muilla maritima* | HM640524 | HM640642 |
| *Muscari aucheri* | HM640510 | HM640628 |
| *Neomarica northiana* | JX903221 | JX903632 |
| *Nerine alta* | JX903159 | JX903569 |
| *Nivenia stokoei* | JX903222 | JX903633 |
| *Nolina bigelovii* | JX903132 | JX903543 |
| *Nothoscordum texanum* | JX903140 | JX903551 |
| *Nypa fruticans* | JX903253 | JX903670 |
| *Odontostomum hartwegii* | JX903231 | JX903647 |
| *Ophiopogon mairei* | KJ745600 | KJ745642 |
| *Ornithogalum shawii* | HM640512 | HM640630 |
| *Polygonatum acuminatifolium* |  |  |
| *Polygonatum arisanense* | -- | KJ745675 |
| *Polygonatum biflorum* | KJ745562 | KJ745725 |
| *Polygonatum cathcartii* | KJ745594 | KJ745753 |
| *Polygonatum cirrhifolium* | KJ745610 | KJ745754 |
| *Polygonatum curvistylum* | KJ745580 | KJ745666 |
| *Polygonatum cyrtonema* | KJ745523 | KJ745644 |
| *Polygonatum desoulavyi* | JX903128 | JX903537 |
| *Polygonatum falcatum* | JX903129 | JX903538 |
| *Polygonatum filipes* |  |  |
| *Polygonatum franchetii* | KJ745558 | -- |
| *Polygonatum grandicaule* | KC704944 | KC704686 |
| *Polygonatum griffithii* | KJ745552 | KJ745752 |
| *Polygonatum hirtellum* | KJ745541 | KJ745758 |
| *Polygonatum hookeri* | KJ745622 | KJ745699 |
| *Polygonatum humile* | KJ745612 | KJ745702 |
| *Polygonatum inflatum* | KJ745524 | KJ745685 |
| *Polygonatum involucratum* |  |  |
| *Polygonatum kingianum* | KJ745517 | KJ745691 |
| *Polygonatum lasianthum* | HM640458 | HM640572 |
| *Polygonatum multiflorum* | -- | KJ745690 |
| *Polygonatum* sp. |  |  |
| *Polygonatum odoratum* | KJ745630 | KJ745674 |
| *Polygonatum oppositifolium* | AB029830 | AB029763 |
| *Polygonatum prattii* Baker | KJ745624 | KJ745712 |
| *Polygonatum pubescens* | KJ745534 | KJ745722 |
| *Polygonatum punctatum* | KJ745554 | KJ745678 |
| *Polygonatum robustum* | KC704960 | KC704714 |
| *Polygonatum roseum* | KJ745631 | KJ745676 |
| *Polygonatum sibiricum* | KJ745581 | KJ745706 |
| *Polygonatum stenophyllum* | KJ745556 | KJ745763 |
| *Polygonatum verticillatum* | KJ745596 | KJ745735 |
| *Polygonatum zanlanscianense* | KJ745608 | KJ745755 |
| *Pancratium canariense* | JX903160 | JX903570 |
| *Pandanus veitchii* | AY952439 | JX903663 |
| *Paradisea minor* | HM640491 | HM640606 |
| *Paramongaia weberbaueri* | JX903161 | JX903571 |
| *Pasithea coerulea* | JX903204 | JX903614 |
| *Pauridia longituba* | JX903210 | JX903620 |
| *Peliosanthes macrostegia* | KJ745599 | KJ745647 |
| *Phoenix dactylifera* | JX903254 | JX903671 |
| *Phormium tenax* | JX903205 | JX903615 |
| *Phragmites australis* | U29900 | AF144575 |
| *Pillansia templemanii* | JX903223 | JX903634 |
| *Pleomele javanica* | JX903130 | JX903541 |
| *Poellnitzia rubiflora* | JX903197 | JX903606 |
| *Ravenea sambiranensis* | JX903255 | JX903672 |
| *Reineckea carnea* | HM640444 | HM640558 |
| *Rhodohypoxis baurii* | HM640540 | HM640658 |
| *Rohdea japonica* | HM640449 | HM640563 |
| *Romulea bulbocodium* | JX903224 | JX903635 |
| *Roscoea cautleoides* | JX903258 | JX903676 |
| *Ruscus aculeatus* | HM640440 | HM640554 |
| *Sansevieria trifasciata* | HM640471 | HM640584 |
| *Scadoxus puniceus* | JX903163 | JX903573 |
| *Scilla scilloides* | HM640514 | HM640632 |
| *Semele androgyna* | HM640442 | HM640556 |
| *Sisyrinchium palmifolium* | JX903225 | JX903636 |
| *Smilax china* | JX903245 | JX903661 |
| *Solenomelus segethii* | JX903226 | JX903637 |
| *Sowerbaea juncea* | JX903189 | JX903597 |
| *Speirantha gardenii* | HM640445 | HM640559 |
| *Spiloxene serrata* | JX903211 | JX903621 |
| *Stawellia dimorphantha* | Z77306 | JX903616 |
| *Stenomesson miniatum* | JX903164 | JX903574 |
| *Stypandra glauca* | JX903206 | JX903617 |
| *Tecophilaea cyanocrocus* | HM640544 | HM640661 |
| *Thereianthus racemosus* | AJ309663 | JX903638 |
| *Theropogon pallidus* | HM640446 | HM640560 |
| *Tigridia immaculata* | JX903227 | JX903639 |
| *Trachycarpus martianus* | JX903256 | JX903673 |
| *Tricalistra ochracea* | AB029839 | AB029777 |
| *Trichopetalum plumosum* | JX903191 | JX903599 |
| *Tricoryne elatior* | JX903207 | JX903618 |
| *Trimezia martinicensis* | JX903228 | JX903640 |
| *Tristagma nivale* | JX903141 | JX903552 |
| *Triteleia peduncularis* | HM640525 | HM640643 |
| *Tulbaghia Simmleri* | JX903143 | JX903554 |
| *Tupistra aurantiaca* | HM640450 | HM640564 |
| *Typha orienthalis* | JX903259 | JX903678 |
| *Ungernia flava* | JX903165 | JX903575 |
| *Urginea epigea* | HM640515 | HM640633 |
| *Vagaria parviflora* | JX903166 | JX903576 |
| *Walleria gracilis* | JX903232 | JX903648 |
| *Watsonia anguta* | JX903229 | JX903641 |
| *Xanthorrhoea media* | JX903234 | JX903650 |
| *Xeronema callistemon* | HM640547 | HM640664 |
| *Yucca filamentosa* | HM640482 | HM640596 |
| *Zephyra elegans* | JX903233 | JX903649 |
| *Zephyranthes simpsonii* | JX903167 | JX903577 |
| *Zingiber mioga* | AF243850 | GU180405 |
